# Discovery of the Natural Bibenzyl Compound Erianin in Dendrobium Inhibiting the Growth and EMT of Gastric Cancer through Downregulating the LKB1-SIK2/3-PARD3 Pathway

**DOI:** 10.3390/ijms25147973

**Published:** 2024-07-21

**Authors:** Xin Wei, Qunshan Liu, Liu Liu, Dan Wang, Jiajia Liu, Qizhi Zhu, Ziming Xu, Qi Chen, Weiping Xu

**Affiliations:** 1 Institute of Intelligent Machines, Hefei Institutes of Physical Science, Chinese Academy of Sciences, Hefei 230031, China; weixin8299@mail.ustc.edu.cn (X.W.); zhu.qz@foxmail.com (Q.Z.); qiqichen421@sina.com (Q.C.); 2 Division of Life Sciences and Medicine, University of Science and Technology of China, Hefei 230026, China; mountainsliu@mail.ustc.edu.cn (Q.L.); lliu0508@163.com (L.L.); gucdan@163.com (D.W.); liujiajia@mail.ustc.edu.cn (J.L.); zimingxu66@163.com (Z.X.); 3 Anhui Provincial Key Laboratory of Tumor Immunotherapy and Nutrition Therapy, Hefei 230001, China

**Keywords:** Erianin, gastric cancer, LKB1, PARD3, epithelial to mesenchymal transition, 5-FU

## Abstract

Erianin, a bibenzyl compound found in dendrobium extract, has demonstrated broad anticancer activity. However, its mechanism of action in gastric cancer (GC) remains poorly understood. *LKB1* is a tumor-suppressor gene, and its mutation is an important driver of various cancers. Yet some studies have reported contradictory findings. In this study, we combined bioinformatics and in vitro and in vivo experiments to investigate the effect and potential mechanism of Erianin in the treatment of GC. The results show that LKB1 was highly expressed in patients’ tumor tissues and GC cells, and it was associated with poor patient prognosis. Erianin could promote GC cell apoptosis and inhibit the scratch repair, migration, invasion, and epithelial–mesenchymal transition (EMT) characteristics. Erianin dose-dependently inhibited the expression of LKB1, SIK2, SIK3, and PARD3 but had no significant effect on SIK1. Erianin also inhibited tumor growth in CDX mice model. Unexpectedly, 5-FU also exhibited a certain inhibitory effect on LKB1. The combination of Erianin and 5-FU significantly improved the anti-tumor efficacy of 5-FU in the growth of GC cells and xenograft mouse models. In summary, Erianin is a potential anti-GC compound that can inhibit GC growth and EMT properties by targeting the LKB1-SIK2/3-PARD3-signaling axis. The synergistic effect of Erianin and 5-FU suggests a promising therapeutic strategy for GC treatment.

## 1. Introduction

Gastric cancer (GC) is a primary epithelial malevolent disease originating from the stomach with multiple stages of development [1]. According to global cancer statistics, GC is the fifth-most common malignant tumor and the fourth cancer-related cause of death worldwide [2]. The morbidity and mortality of GC rank third among all types of tumors in China [3]. The onset of GC is affected by various risk factors, including obesity, *Helicobacter* pylori (H. pylori) infection, gastrointestinal microbiota, smoking, alcohol consumption, radiation or viral infection, and unhealthy eating habits and lifestyle [3,4,5,6,7]. Surgery is the primary treatment to eradicate GC lesions [8]. However, since most GC patients are diagnosed at a late stage, surgery alone is sometimes insufficient. The use of perioperative chemotherapy can increase the survival probability of operable GC patients [9]. Neoadjuvant triple chemotherapy, including the combination of docetaxel, oxaliplatin, cisplatin, and 5-fluorouracil (5-FU), is a common chemotherapy strategy for GC patients before surgery and has achieved good clinical results [10]. Although the survival rate of GC has improved with the development of early screening, diagnostic, and therapeutic technologies, GC still maintains a high mortality rate and is an important component of the global disease burden [11,12]. It is still urgent to actively search for therapeutic targets and drugs for GC.

Natural products have unique advantages in disease treatment and have received widespread attention from drug developers in recent years. Erianin, a bibenzyl compound in the traditional Chinese medicine dendrobium extract, shows inhibitory effects on various tumors [13]. Specifically, it can exert anti-cancer effects by inhibiting the viability of tumor cells, arresting the cell cycle, and promoting cell apoptosis [14]. However, previous studies have primarily focused on the effect and development potential of Erianin against liver cancer, lung cancer, bladder cancer, etc. [15,16,17], while the research on Erianin for GC is still very limited [18]. Therefore, exploring the anti-GC effect of Erianin and elucidating its possible mechanism is of great significance and has broad prospects for the application of Erianin and the treatment of GC.

Liver kinase B1 (LKB1), also referred to as serine/threonine (Ser/Thr) kinase 11 (STK11), is an important tumor-suppressor gene. Mutations in *LKB1* are believed to be closely associated with the development of various cancers, such as carcinoma of the lungs [19], hepatoma [20], cervical cancer [21], pancreatic cancer [22], and melanoma [23]. However, most current studies have pointed out that a low expression of *LKB1* is a prominent reason for the outbreaks and progress of many cancers. However, in most sporadic tumors, *LKB1* mutations are rare [24], with the exception of non-small cell lung cancer (NSCLC), where *LKB1* mutations can reach 15–35% [25]. Researchers have conducted extensive studies on the role of LKB1 in NSCLC, showing that LKB1 is involved in processes such as tumor differentiation, metastasis, immune response, and chemotherapy resistance [26,27]. Interestingly, some studies have reported that restoring the level of LKB1 in NSCLC cell lines did not lead to cell apoptosis but instead increased the expression of the multidrug resistance-related protein 1 (MDR1), resulting in reduced cell sensitivity to paclitaxel [28]. In a study of clinical samples of GC, it was found that the mutation rate of *LKB1* in tumor tissues was low, indicating that somatic mutations of *LKB1* are uncommon in sporadic GC [29]. The mechanism of action of LKB1 in GC remains to be further explored. Salt-inducible kinases (SIKs) are classified under the AMP-activated protein kinase family and include three subtypes (SIK1, SIK2, and SIK3) with different functions. As a direct downstream gene of *LKB1*, under normal circumstances, SIK1 and SIK2 are mainly involved in body metabolism, while SIK3 promotes the occurrence of cancer by interacting with the mTOR complex. The dysregulation of the SIK function is associated with the development of several cancers, including breast, gastric, and ovarian cancer. According to most reported research, SIK1 is considered a tumor suppressor, while SIK2 and SIK3 are often associated with tumor promotion [30]. Studies have reported that the use of SIK inhibitors sensitizes ovarian cancer cells to paclitaxel [31], and that the specific knockout of *SIK2* inhibited the migration and proliferation of osteosarcoma cells [32]. However, the function of SIKs in some tumors seems to be dual-sided, preventing us from decisively defining SIKs simply as tumor-promoting or tumor-suppressing genes. Previous studies have shown that the loss of Partition defective 3 (PARD3) impairs the migration ability of *Drosophila* ovarian border cells [33]. However, subsequent studies have shown that PARD3 is significantly upregulated in various cancers, including liver [34], ovarian [35], prostate [36], and colorectal cancer [37], and it plays a role in maintaining growth, promoting proliferation, and facilitating metastasis. A report has indicated that in NMuMG cells, SIK can directly act on PARD3 and negatively regulate its expression [38]. However, whether and how SIKs regulate PARD3 in GC cells is still unknown.

In preliminary experiments, we found that Erianin significantly inhibited the growth of GC cells and downregulated the protein expression of LKB1. To explore the anti-GC mechanism of Erianin and the regulatory pathways related to LKB1, this study researched clinical samples and bioinformatics and confirmed the effect and mechanism of Erianin in treating GC both in vivo and in vitro. This may provide a research basis for the discovery of candidate compounds for anti-GC.

## 2. Results

### 2.1. LKB1 Is Highly Expressed in Gastric Cancer and Associated with Poor Patient Prognosis

**Figure 1:**
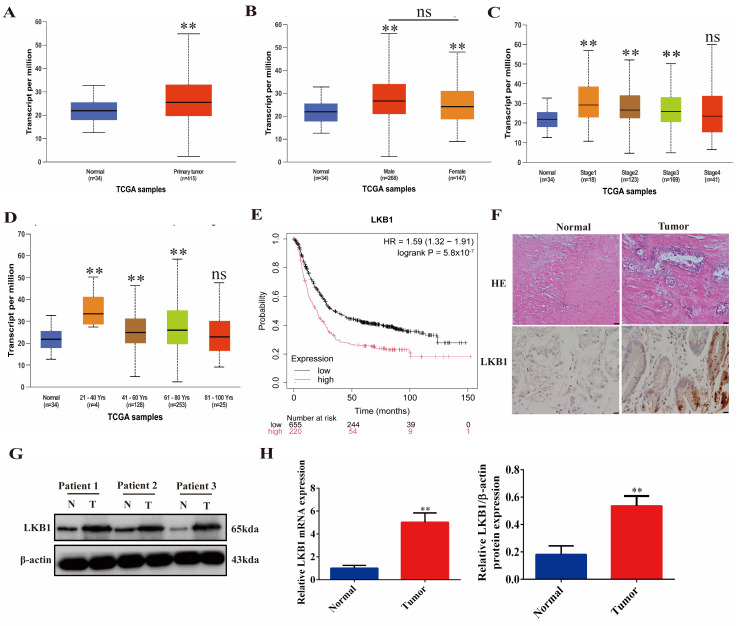
LKB1 is highly expressed in GC patients and is associated with poor prognosis. Analysis of *LKB1* expression levels in normal gastric tissue and all primary GCs using the UALCAN portal (**A**), with subgroup analysis based on patient gender (**B**), tumor stage (**C**), and patient age (**D**). (**E**) High *LKB1* expression was associated with poor survival in GC patients. (**F**) IHC staining of LKB1 in paracancerous tissues and GC tissues of clinical patients (HE: 200×, IHC: 400×). (**G**,**H**) Expression levels of *LKB1* protein and gene in paracancerous tissues and GC tissues of clinical patients (N: Normal, T: Tumor, *n* = 3). ** *p* < 0.01 indicates statistical difference compared to the Normal group.

An analysis of the UALCAN database based on TCGA samples showed that, compared with normal samples, the expression of *LKB1* in GC samples was greatly increased (*p* < 0.01, Figure 1A). In both male and female patients, *LKB1* was highly expressed compared with the normal group (*p* < 0.01), but the difference in *LKB1* expression between genders was not significant (Figure 1B). *LKB1* continued to maintain a high expression from Stage 1 to Stage 3 compared with the normal group (*p* < 0.01). However, as the stage progressed, the level of LKB1 gradually decreased. By Stage 4, the *LKB1* level was not significantly different from the normal group (Figure 1C). The expression of *LKB1* was also related to the patient’s age. In GC patients aged between 21 and 40 years old, *LKB1* was significantly more expressed than in the normal group (*p* < 0.01) and was higher than the levels of *LKB1* in patients aged between 41 and 80 years old. However, the *LKB1* level of GC patients in the 81- to 100-year-old range was not significantly different from the normal group (Figure 1D). An analysis of the prognostic contribution of *LKB1* to GC patients through the Kaplan–Meier Plotter database showed that high expression levels of *LKB1* predict shorter patient survival (*p* < 0.01, Figure 1E). An immunohistochemical (IHC) analysis of GC tumor tissues and adjacent normal tissues extracted from clinical patients proved that LKB1-positive staining increased significantly in GC tissues (Figure 1F). Protein and gene expression analyses of patients’ normal and GC tissues showed that, compared with adjacent tissues, the gene and protein levels of *LKB1* in tumor sites were significantly increased (Figure 1G,H). These results indicate that a high expression of LKB1 is associated with the occurrence and progression of GC.

### 2.2. LKB1 Is Closely Related to SIKs and PARD3, and They Are Highly Expressed in Gastric Cancer

**Figure 2:**
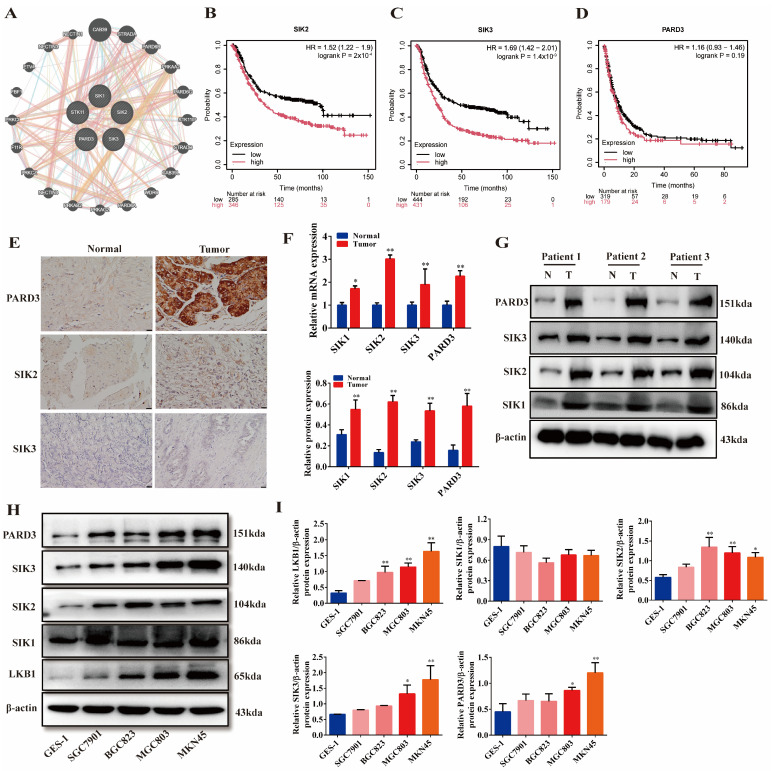
Association of LKB1 with SIK1−3 and PARD3 and their roles in GC. (**A**) The gene association network of *LKB1*, *SIK1*−*3*, and *PARD3* was constructed using the GeneMANIA data portal. (**B**) Relationship between *SIK2* expression and the survival curve of GC patients. (**C**) Relationship between *SIK3* expression and the survival curve of GC patients. (**D**) The relationship between *PARD3* and the survival curve of GC patients. (**E**) IHC staining of SIK2, SIK3, and PARD3 in paracancerous and GC tissues of GC patients (400×). (**F**,**G**) Differences in gene and protein expression levels of *SIK2*, *SIK3*, and *PARD3* in paracancerous and GC tissues of GC patients (*n* = 3). (**H**,**I**) Differential protein expression of LKB1, SIKs, and PARD3 in four GC cell lines and GES-1. * *p* < 0.05, ** *p* < 0.01 indicates statistical difference compared to the normal group (**F**) or GES-1 group (**I**).

Using the GeneMANIA database, an association network was constructed between *LKB1* (*STK11*), *SIK1*, *SIK2*, *SIK3*, and *PARD3*. The analysis revealed that *LKB1* had a close connection with *SIKs* and *PARD3* (Figure 2A). Kaplan–Meier Plotter analysis showed that a high expression of *SIK2* and *SIK3* was associated with a shorter survival of GC patients (Figure 2B,C, *p* < 0.01). However, a high expression of *PARD3* was not significantly associated with poor patient survival (Figure 2D, *p* > 0.05). IHC staining of clinical patient tissues demonstrated that SIK2, SIK3, and PARD3 were highly expressed in GC tissues compared to normal tissues (Figure 2E). Protein and gene expression analyses in patient samples revealed that *SIK1*, *SIK2*, *SIK3*, and *PARD3* were all highly expressed in tumor tissues compared to adjacent normal tissues (Figure 2F,G, *p* < 0.01). Compared to the normal gastric epithelial cell GES-1, LKB1 was highly expressed to varying degrees in the four GC cell lines. The expression of LKB1 was significantly higher in MGC803 and MKN45 cells (Figure 2H,I, *p* < 0.01). Consistent with this, SIK2, SIK3, and PARD3 were also significantly over-expressed in GC cell lines (*p* < 0.01). MGC803 cells and MKN45 cells were selected for subsequent experiments. Interestingly, there was no significant difference in the SIK1 expression between GES-1 and GC cell lines, suggesting that SIK1 may not be directly involved in the regulation of LKB1-mediated-signaling pathways in GC.

### 2.3. Erianin Inhibits GC Cell Viability and Induces Apoptosis

**Figure 3:**
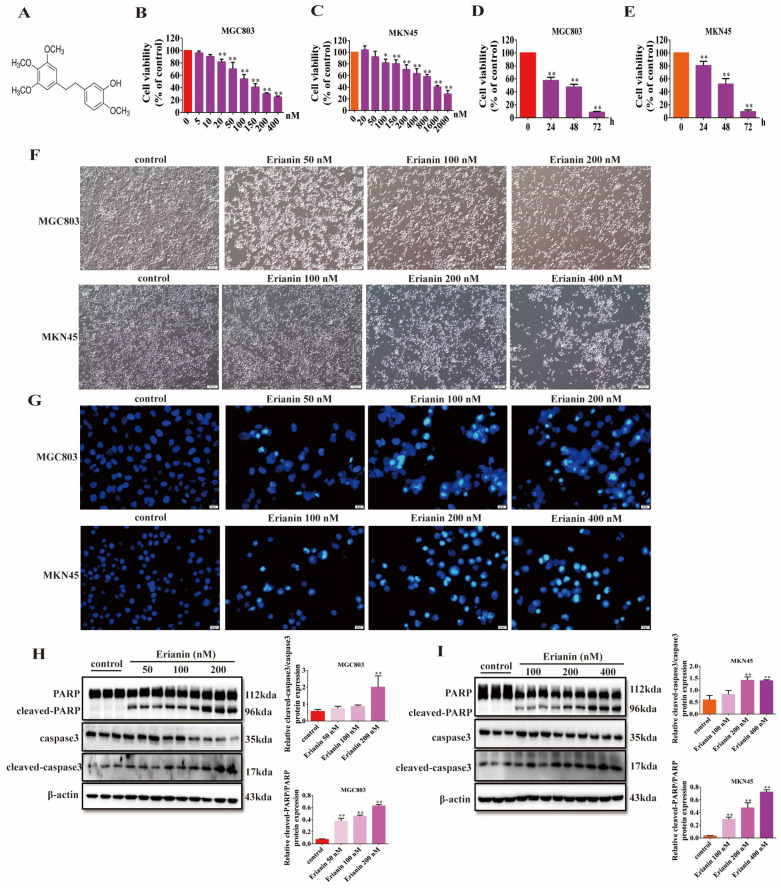
Erianin inhibits the viability of GC cells and induces cell apoptosis. (**A**) The chemical structural formula of Erianin. (**B**) Effects of Erianin treatment on the viability of MGC803 cells at different concentrations. (**C**) Effects of different concentrations of Erianin on cell viability of MKN45 cells. (**D**) Effect of 100 nM Erianin treatment on the viability of MGC803 cells for 24, 48, and 72 h. (**E**) Effects of 200 nM Erianin treatment on the viability of MKN45 cells for 24, 48, and 72 h. (**F**) Effects of different concentrations of Erianin on the cell morphology of MGC803 cells and MKN45 cells for 24 h of treatment (scale bar = 100 μm). (**G**) Hoechst 33342 staining of MGC803 cells and MKN45 cells treated with different concentrations of Erianin for 24 h (scale bar = 20 μm). (**H**) Erianin increased the expression of apoptosis-related proteins in MGC803 cells in a dose-dependent manner. (**I**) Erianin increased the expression of apoptosis-related proteins in MKN45 cells in a dose-dependent manner. * *p* < 0.05, ** *p* < 0.01 indicates statistical difference compared to the control group.

The chemical structure of Erianin is shown in Figure 3A. Erianin inhibited the viability of MGC803 cells in a dose-dependent manner (Figure 3B). When MGC803 cells were treated with 100 nM Erianin, their viability decreased over time (Figure 3D). A similar time-dependent reduction in cell viability was observed in MKN45 cells, although they were less sensitive to Erianin, requiring a higher concentration (200 nM) to achieve a comparable decrease in viability (Figure 3C,E). Morphological analysis revealed that treatment with Erianin (50–400 nM) for 24 h significantly altered the appearance of both MGC803 and MKN45 GC cells and inhibited their proliferation (Figure 3F). Hoechst 33342 staining further demonstrated that Erianin increased the number of apoptotic GC cells in a dose-dependent manner (Figure 3G). At the protein level, Erianin treatment led to a dose-dependently increase in the expression of cleaved-PARP and cleaved-caspase3, markers of apoptosis in the GC cells (Figure 3H,I). These results indicate that Erianin effectively inhibits the viability and proliferation of GC cells and promotes their apoptosis in a concentration-dependent manner.

### 2.4. Erianin Inhibits the Expression of LKB1, SIK2/3 and PARD3 in GC Cells

**Figure 4:**
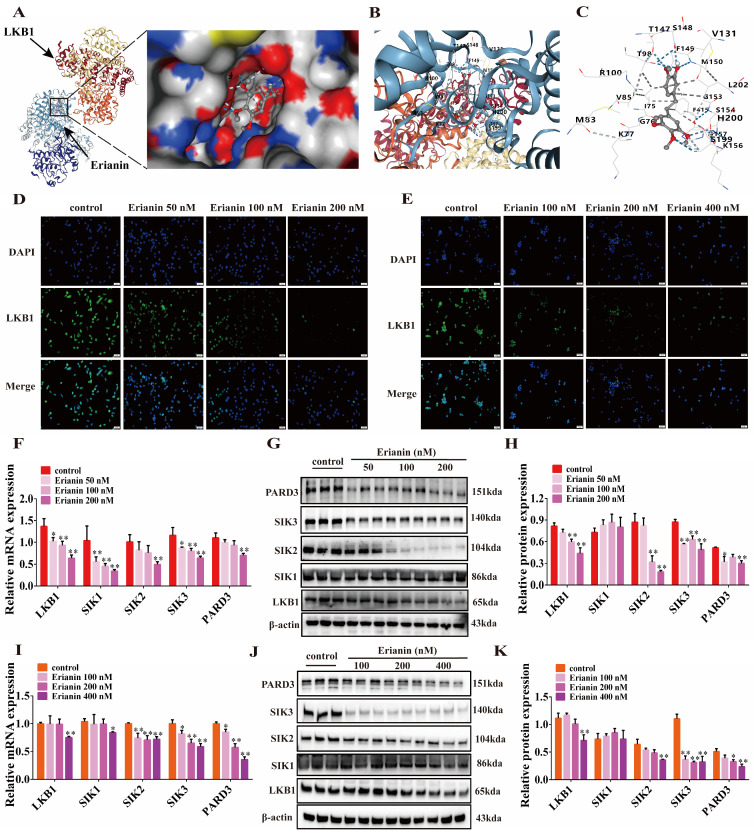
Erianin inhibits the expression of LKB1, SIK2/3 and PARD3, but not SIK1, in GC cells. (**A**) Molecular docking conformation of Erianin and LKB1 (PDB ID: 2WTK). Arrows indicated the locations of LKB1 and Erianin. The black box magnifies the 3D view of the docking cavity of Erianin and LKB1. (**B**,**C**) Distribution of amino acid residues around the binding pocket of Erianin and LKB1 and the chemical bonds formed. The oxygen atom on Erianin formed hydrogen bonds with Ser148, Ser199, Ser154, Thr147, Met150 and Thr98. The oxygen atom on Erianin formed a weak hydrogen bond with Ser199, and the carbon atom formed a weak hydrogen bond with Asp157. The carbon atom on the benzene ring of Erianin formed hydrophobic interactions with Leu202 and Ile75. The carbon atom on the C=C connecting the two benzene rings in Erianin formed a hydrophobic interaction with Phe415. (**D**,**E**) Erianin inhibited the fluorescence intensity of LKB1 in MGC803 and MKN45 cells (scale bar = 50 μm). (**F**) Effects of treating MGC803 cells with an Erianin concentration gradient on the expression of *LKB1*, *SIK1*−*3*, and *PARD3* genes. (**G**,**H**) Effects of treating MGC803 cells with an Erianin concentration gradient on the expression of LKB1, SIK1−3, and PARD3 proteins. (**I**) Effects of treating MKN45 cells with an Erianin concentration gradient on the expression of *LKB1*, *SIK1*−*3*, and *PARD3* genes. (**J**,**K**) Effects of treating MKN45 cells with an Erianin concentration gradient on the protein expression of LKB1, SIK1−3, and PARD3. * *p* < 0.05, ** *p* < 0.01 indicates statistical difference compared to the control group.

The CB-Dock2 online docking analysis revealed that Erianin had a good docking potential with a spatial of 3436 Å^3^. In its optimal docking conformation (docking center: X = −50, Y = −9, Z = 19), the affinity was calculated to be −8 kcal/mol (Figure 4A), with the docking size measuring: x = 27 Å, y = 31 Å, z = 21 Å. Erianin formed hydrogen bonds or hydrophobic interaction with the amino acid residues Ser154, Leu202, Met150, Ser148, Ile75, Phe415, and Ser199 surrounding the binding pocket of LKB1 (Figure 4B,C). Consistent with the docking data, Erianin significantly inhibited the fluorescent expression of LKB1 in both MGC803 and MKN45 cells (Figure 4D,E). In MGC803 cells, the gene expression of *LKB1* was reduced in a concentration-dependent manner upon treatment with 50, 100, and 200 nM Erianin, with a significant decrease observed at 200 nM (*p* < 0.05). Additionally, the gene expression of the LKB1 downstream targets *SIK2*, *SIK3*, and *PARD3* were all inhibited by Erianin to varying degrees (Figure 4F). At the protein level, Erianin inhibited the expression of LKB1 and PARD3 in a dose-dependent manner. Interestingly, the SIK family proteins showed differential responses to Erianin treatment. While SIK1 protein levels were largely unaffected, SIK2 and SIK3 were significantly inhibited at 100 nM Erianin (Figure 4G–K). These findings suggest that Erianin’s regulation of GC cells is primarily mediated through the inhibition of SIK2 and SIK3, rather than SIK1.

### 2.5. Erianin Inhibits GC Cell Migration, Invasion, and EMT Properties

**Figure 5:**
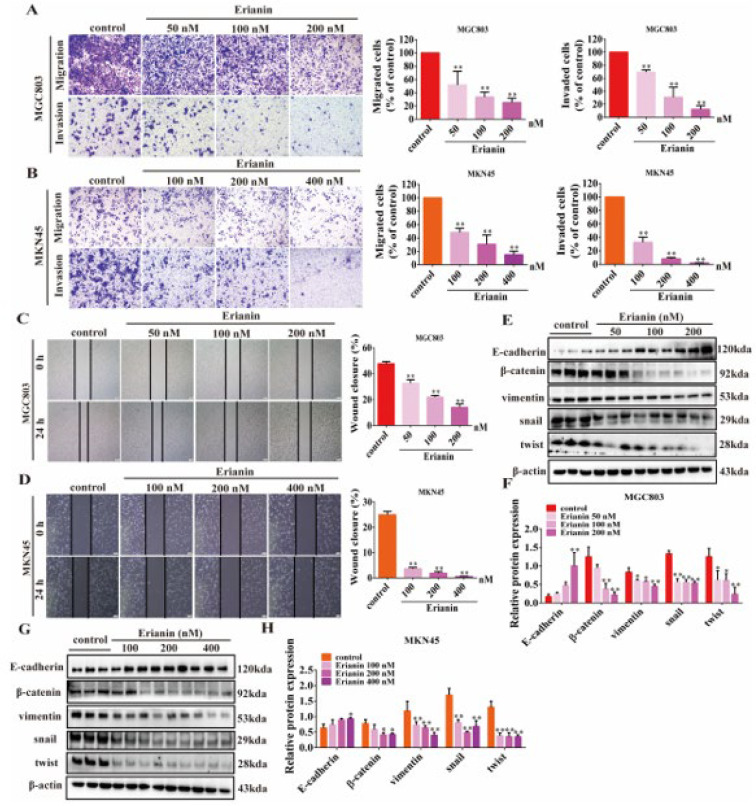
Erianin inhibits migration, invasion, and EMT properties in GC cells. (**A**) Effects of different concentrations of Erianin on transwell migration and invasion ability of MGC803 cells (scale bar =100 μm). (**B**) Effects of different concentrations of Erianin on transwell migration and invasion ability of MKN45 cells (scale bar =100 μm). (**C**) Effect of Erianin concentration on wound healing ability of MGC803 cells (scale bar =200 μm). (**D**) Effect of Erianin concentration on wound healing ability of MKN45 cells (scale bar =200 μm). (**E**,**F**) Erianin reversed the expression of EMT marker proteins in MGC803 cells. (**G**,**H**) Erianin reversed the expression of EMT marker proteins in MKN45 cells. * *p* < 0.05, ** *p* < 0.01 indicates statistical difference compared to the control group.

To evaluate the functional impact of Erianin on GC cells, we examined the migration, invasion, and scratch wound repair abilities of MGC803 and MKN45 cells after Erianin treatment. The results showed that Erianin effectively inhibits migration and invasion (Figure 5A,B), as well as wound healing (Figure 5C,D), in both cell lines in a concentration-dependent manner. Furthermore, Erianin treatment increased the protein levels of E-cadherin in a dose-dependent fashion. Conversely, the expression of β-catenin, vimentin, snail, and twist was reduced by Erianin in a concentration-dependent manner (Figure 5E–H). These findings indicate that Erianin could significantly inhibit the EMT characteristics of GC cells.

### 2.6. Regulatory Relationship of LKB1-SIK2/3-PARD3

**Figure 6:**
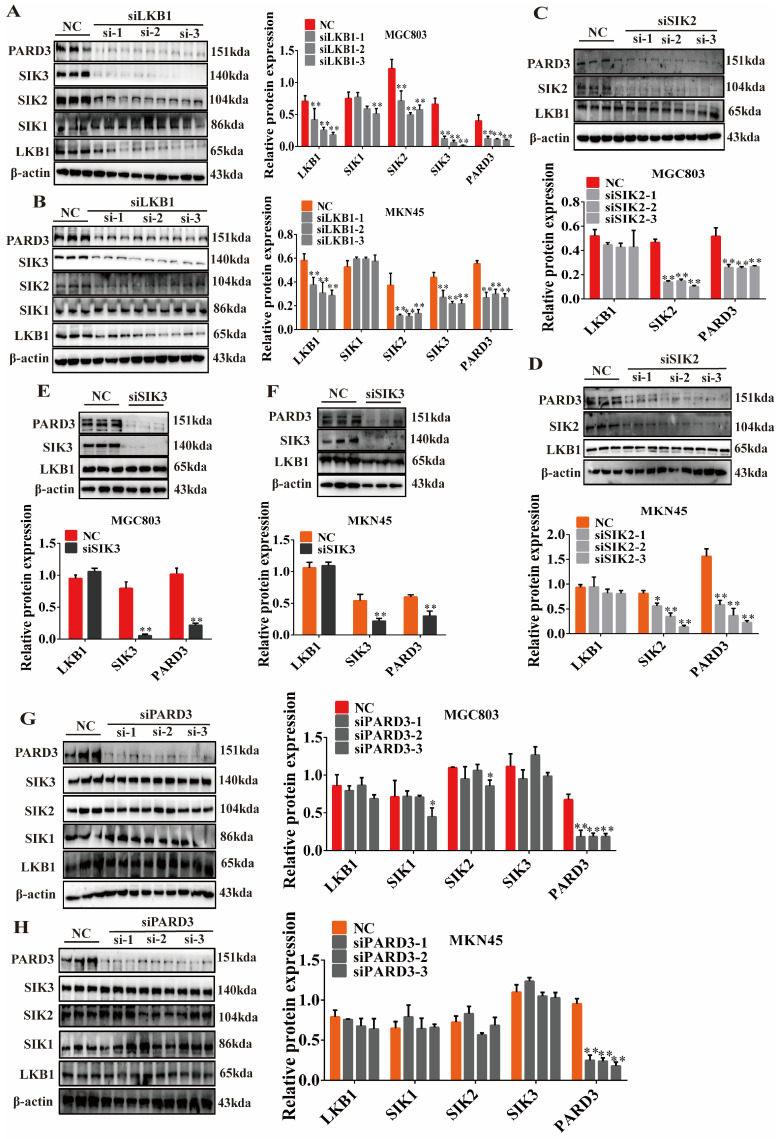
Silencing the expression of LKB1, SIKs, and PARD3 and their interaction. (**A**,**B**) Effects of silencing LKB1 expression on the expression of SIK1−3 and PARD3 in GC cells. (**C**,**D**) Effects of silencing SIK2 expression on the expression of LKB1 and PARD3 in GC cells. (**E**,**F**) Effects of silencing SIK3 expression on the expression of LKB1 and PARD3 in GC cells. (**G**,**H**) Effects of silencing PARD3 expression on the expression of LKB1 and SIK1−3 in GC cells. * *p* < 0.05, ** *p* < 0.01 indicates statistical difference compared to the NC group.

The previous results showed that Erianin had a good affinity with LKB1 and inhibited the protein expression of LKB1, SIK2/3, and PARD3. To further understand the upstream and downstream regulatory relationships among these proteins, we transiently knocked down *LKB1*, *SIK2*, *SIK3*, and *PARD3* in dividually MGC803 cells and MKN45 cells. The results demonstrated that silencing LKB1 (*siLKB1*) inhibited the expression of LKB1 in GC cells while also reducing the levels of SIK2, SIK3, and PARD3 but had no obvious effect on SIK1 (Figure 6A,B). This was consistent with the previous findings, indicating a limited regulatory effect of LKB1 on SIK1 in GC cells. Furthermore, knocking down SIK2 (*siSIK2*) or SIK3 (*siSIK3*) did not affect LKB1 expression but inhibited the expression of PARD3 (Figure 6C–F). Silencing PARD3 (*siPARD3*), on the other hand, did not significantly impact the levels of LKB1, SIK1, SIK2, or SIK3 (Figure 6G,H). Collectively, these results indicated that reduced LKB1 levels induced by Erianin inhibited the expression of SIK2/3, which subsequently led to the downregulation of PARD3. This preliminary analysis helps elucidate the inhibitory mechanism of Erianin on GC cells mediated through the LKB1-signaling axis.

### 2.7. Erianin Inhibits Tumor Growth in CDX Mice

**Figure 7:**
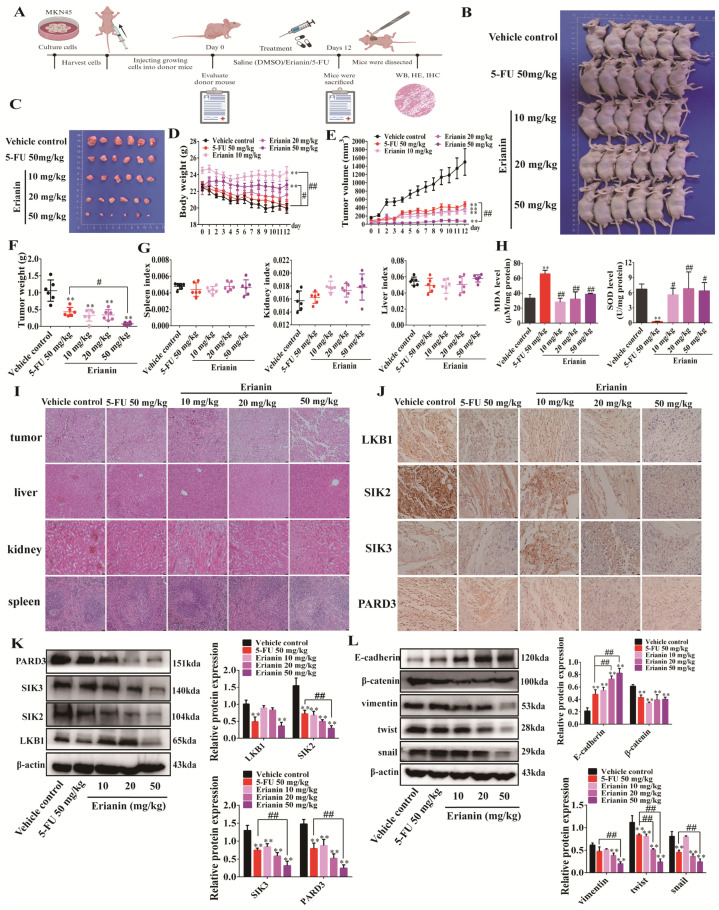
Erianin inhibits tumor growth and the expression of LKB1, SIK2, SIK3, and PARD3 in the CDX mouse model. (**A**) Schematic diagram of the CDX model construction and drug administration treatment strategy. (**B**) Axillary tumor growth of mice in the five groups. (**C**) The size of isolated tumors from mice in the five groups. (**D**) Body weight changes of mice in the five groups during the administration period. (**E**) Changes in tumor volume during the administration of mice in the five groups. (**F**) Tumor mass in the five groups of mice. (**G**) Liver, spleen, and kidney index of mice in each group. (**H**) Differences in liver MDA and SOD levels of mice in the five groups. (**I**) Representative photomicrographs of pathological staining of tumors, liver, spleen, and kidney tissues of mice in the five groups (200×). (**J**) IHC staining results of LKB1, SIK2, SIK3, and PARD3 in mouse tumor tissues in the five groups (400×). (**K**) Changes in protein expression of LKB1, SIK2, SIK3, and PARD3 in tumor tissues of mice in the five groups. (**L**) Changes in the expression of EMT marker proteins in tumor tissues of mice in the five groups. ** *p* < 0.01 indicates statistical difference compared to the vehicle control group. ^#^ *p* < 0.05, ^##^ *p* < 0.01 indicates statistical difference compared to the 5-FU 50 mg/kg group.

To verify the anti-tumor effect of Erianin in vivo, we constructed a cell-derived xenograft (CDX) mouse model by injecting MKN45 cells and administered Erianinor 5-FU via intraperitoneal injection (Figure 7A). The results showed that Erianin inhibited tumor growth in a dose-dependent manner (Figure 7B,C), but unlike 5-FU, it did not significantly affect the body weight of mice (Figure 7D). Erianin treatment led to a dose-dependent reduction in tumor volume and size, and the 50 mg/kg Erianin group exhibited stronger anti-tumor effects than 5-FU (Figure 7E,F). To evaluate the organ toxicity of intraperitoneal of Erianin administration, we examined the liver, spleen, and kidney indices of the mice. The results showed no significant differences among the groups, indicating that the Erianin dosage used in this study was relatively safe (Figure 7G), which was further confirmed by the H&E staining (Figure 7I). We also analyzed the effects of drugs on oxidative stress in the mouse livers. While 5-FU increased MDA levels and reduced SOD levels, each Erianin group showed no significant changes compared to the control group (Figure 7H), suggesting that Erianin may be safer than 5-FU. IHC and western blot analyses revealed that Erianin inhibited the expression of LKB1, SIK2, SIK3, and PARD3 in a dose-dependent manner in the mouse tumor tissues, and 5-FU also significantly reduced the levels of these proteins (Figure 7J,K). Additionally, Erianin increased the expression of E-cadherin in a dose-dependent manner while decreasing the levels of β-catenin, vimentin, twist, and snail (Figure 7L), indicating that Erianin could also inhibit the EMT properties of tumors in vivo.

### 2.8. 5-FU Inhibits the Expression of LKB1, SIK2/3, and PARD3 in GC Cells

**Figure 8:**
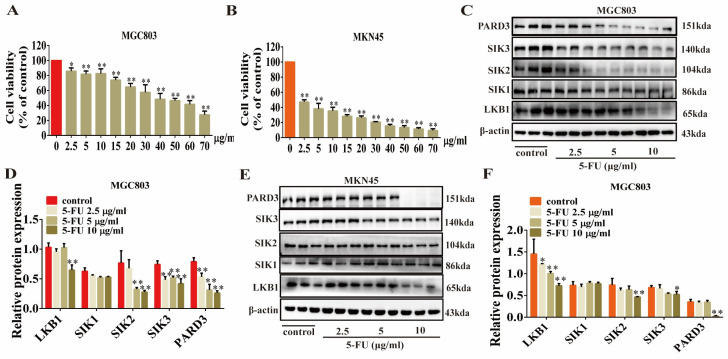
5-FU inhibits the expression of LKB1, SIK2, SIK3, and PARD3 in GC cells. (**A**) CCK8 assay showing the inhibitory effect of different concentrations of 5-FU on MGC803 cells. (**B**) CCK8 detected the inhibitory effect of different concentrations of 5-FU on MKN45 cells. (**C**,**D**) 5-FU inhibited the protein expression of LKB1, SIK2, SIK3 and PARD3 in MGC803 cells in a dose-dependent manner. (**E**,**F**) 5-FU inhibited the proteins expression of LKB1, SIK2, SIK3 and PARD3 in MKN45 cells in a dose-dependent manner. * *p* < 0.05, ** *p* < 0.01 indicates statistical difference compared to the control group.

The results of animal experiments showed that 5-FU had a certain effect on the expression of the LKB1 protein, which led us to consider the possibility of a combined drug strategy using 5-FU and Erianin. We first screened the concentration of 5-FU administered to MGC803 and MKN45 cells. The results indicated that MKN45 cells were more sensitive to 5-FU than MGC803 cells (Figure 8A,B). Considering these findings, we selected 2.5, 5, and 10 μg/mL as the doses for the study. Under this gradient concentration treatment, we found that 10 μg/mL 5-FU had a significant inhibitory effect on the expression of LKB1 in the two types of GC cells (*p* < 0.01). In MGC803 cells, 5-FU inhibited the levels of SIK2, SIK3, and PARD3 (*p* < 0.01) but had no significant effect on SIK1 (Figure 8C,D), which was consistent with the effect of Erianin. In MKN45 cells, 5-FU had a smaller inhibitory effect on SIK2 and SIK3 (*p* < 0.05) but almost completely inhibited PARD3 at 10 μg/mL (Figure 8E,F).

### 2.9. 5-FU Combined with Erianin Jointly Inhibits the Expression of LKB1-SIK2/3-PARD3 Axis

**Figure 9:**
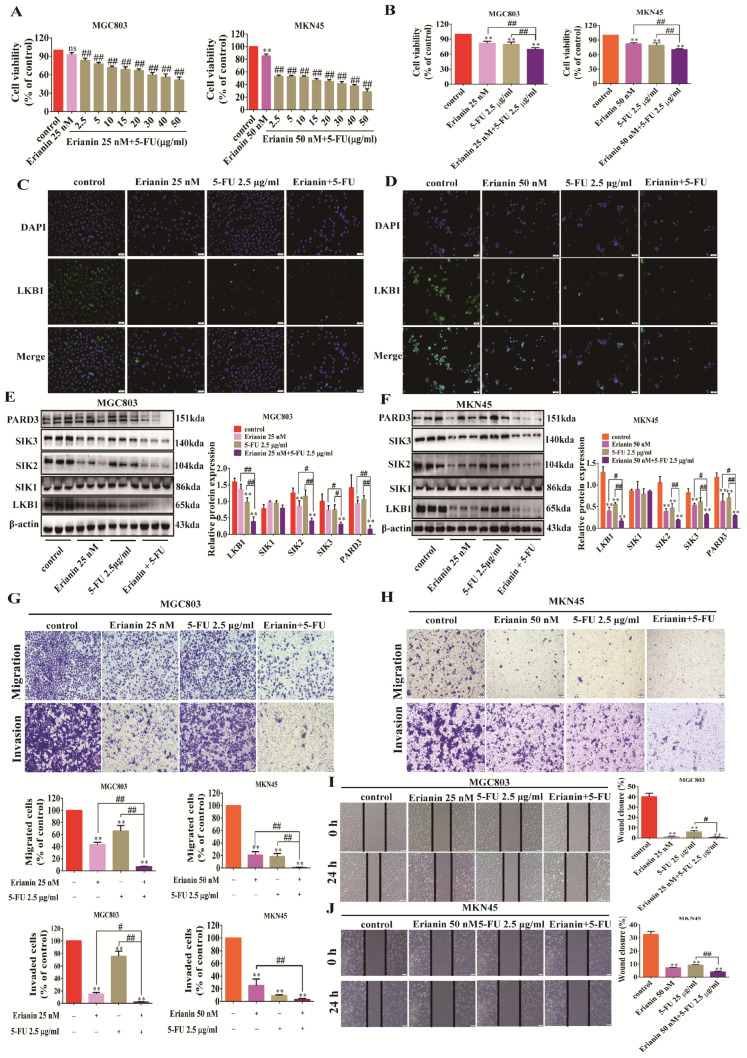
5−FU combined with Erianin inhibits the expression of LKB1, SIK2/3, and PARD3 in GC cells and the migration and invasion ability of cells. (**A**) CCK8 examined the inhibitory effect of Erianin 25 nM or Erianin 50 nM combined with different concentrations of 5-FU on the cell viability of MGC803 or MKN45 cells. (**B**) Inhibitory effects of Erianin at 25 nM combined with 2.5 μg/mL 5-FU, and Erianin at 50 nM combined with 2.5 μg/mL 5-FU on the cell viability of MGC803 or MKN45. (**C**) Erianin at 25 nM combined with 5-FU at 2.5 μg/mL inhibited the fluorescent expression of LKB1 in MGC803 cells (scale bar = 50 μm). (**D**) 50 nM Erianin combined with 2.5 μg/mL 5-FU inhibited the fluorescent expression of LKB1 in MKN45 cells (scale bar = 50 μm). (**E**) Erianin combined with 5-FU inhibited the protein expression of LKB1, SIK1−3, and PARD3 in MGC803 cells. (**F**) Erianin combined with 5-FU inhibited the protein expression of LKB1, SIK1−3, and PARD3 in MKN45 cells. (**G**) Erianin combined with 5-FU inhibited the migration and invasion of MGC803 cells (scale bar = 100 μm). (**H**) Erianin combined with 5-FU inhibited the migration and invasion of MKN45 cells (scale bar =100 μm). (**I**) Erianin combined with 5-FU inhibited wound healing of MGC803 cells (scale bar = 100 μm). (**J**) Erianin combined with 5-FU inhibited wound healing of MKN45 cells (scale bar = 200 μm). * *p* < 0.05, ** *p* <0.01 indicates statistical difference compared to the control group. ^#^ *p* < 0.05, ^##^ *p* < 0.01 indicates statistical difference compared to the Erianin+5FU group.

First, we chose to halve the concentration of Erianin and examined the inhibition of cell viability of GC cells by different combinations under a combination administration strategy, with 5-FU concentrations ranging from 2.5–50 μg/mL. The results showed that 2.5 μg/mL 5-FU combined with 25 nM Erianin could considerably inhibit the viability of MGC803 cells, while 2.5 μg/mL 5-FU combined with 50 nM Erianin could notably inhibit the viability of MKN45 cells (Figure 9A,B). Compared to 5-FU or Erianin alone, the combination of 5-FU and Erianin could significantly inhibit the fluorescence and protein expression of LKB1 both in MGC803 and MKN45 cells (Figure 9C–F). Furthermore, the combination also significantly inhibited the expression of SIK2, SIK3, and PARD3 (Figure 9E,F). In terms of cell function, the combination of 5-FU and Erianin inhibited cell migration and invasion better than either drug administration alone (Figure 9G,H). Similarly, the combination had a higher inhibition rate on the wound-healing ability compared to either drug alone (Figure 9I,J).

### 2.10. Erianin Combined with 5-FU Inhibits Tumor Growth in CDX Mice

**Figure 10:**
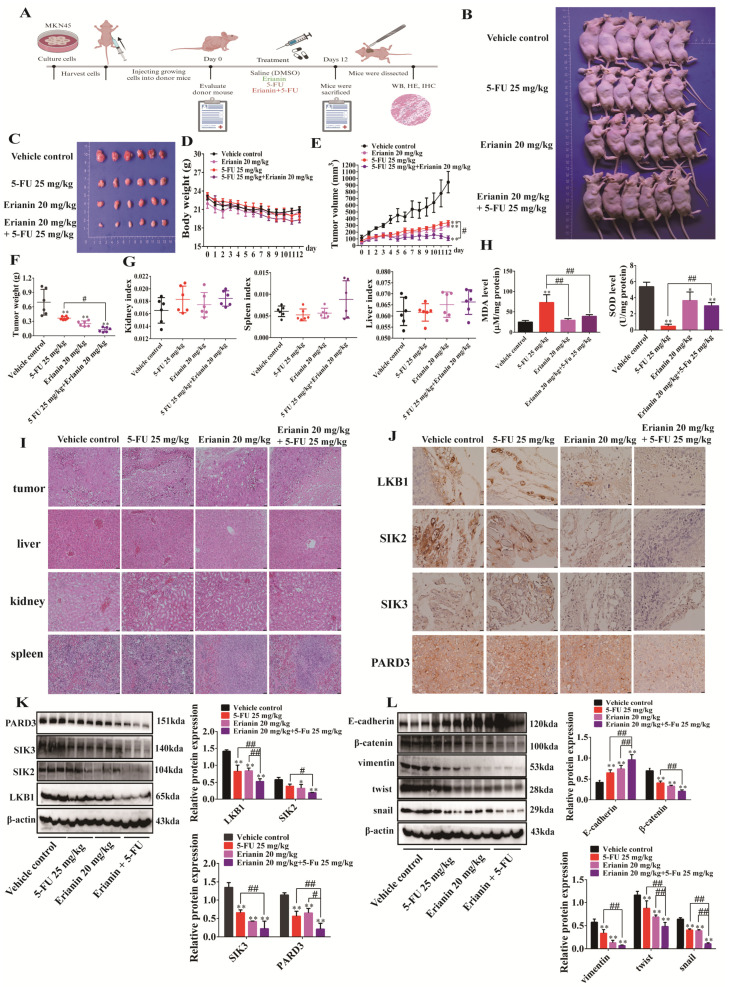
Erianin combined with 5-FU inhibits tumor growth in CDX mouse model. (**A**) Schematic diagram of the CDX mouse model construction and the administration strategy for Erianin combined with 5-FU. (**B**) Growth of subcutaneous tumors in mice. (**C**) Photographs of subcutaneous tumors in mice. (**D**) Body weight change curve of mice in each group during the administration period. (**E**) Tumor volume change curves of mice in the four groups during the administration period. (**F**) Tumor weight of mice in the four groups. (**G**) Liver, spleen, and kidney indices of mice in the four groups. (**H**) Differences in liver MDA and SOD contents among the four groups of mice. (**I**) H&E staining of tumors, liver, spleen, and kidneys of mice in the four groups (200×). (**J**) IHC staining of LKB1, SIK2, SIK3, and PARD3 in tumor tissues from mice in the four groups (400×). (**K**) Protein expression of LKB1, SIK2, SIK3, and PARD3 in tumor tissues from mice in the four groups. (**L**) Expression of EMT marker proteins in tumor tissues from mice in the four groups. * *p* < 0.05, ** *p* < 0.01 indicates statistical difference compared to the Vehicle control group. ^#^ *p* < 0.05, ^##^ *p* < 0.01 indicates statistical difference compared to the Erianin+5-FU group.

Consistent with previous models, we constructed the CDX model by subcutaneously injecting MKN45 cells into mice. The dosage regimen was as follows: 5-FU at 25 mg/kg every 3 days, and Erianin at 20 mg/kg daily. The combined dosing strategy was 25 mg/kg5-FU every 3 days plus 20 mg/kg Erianin daily (Figure 10A). Compared to the single administration of 5-FU or Erianin, their combined administration inhibited the growth of subcutaneous tumors more significantly (Figure 10B,C). There were no significant differences in body weight changes between groups (Figure 10D). The combination of 5-FU and Erianin inhibited the volume and weight of mouse tumors more significantly, with a better effect than either drug alone (Figure 10E,F). There were also no significant differences in liver, spleen, and kidney indices between groups (Figure 10G). Interestingly, the addition of Erianin inhibited the increase in MDA and decrease in SOD in mouse livers caused by 5-FU (Figure 10H). Importantly, no significant pathologic changes were observed in the liver, spleen, and kidneys of mice in each administration group (Figure 10I), indicating that the dosage regimen did not cause obvious toxicity. Compared to the individual drug, Erianin combined with 5-FU notably inhibited the expression of LKB1, SIK2, SIK3, and PARD3 in mouse tumor tissues (Figure 10J,K). Moreover, the combination increased the expression of E-cadherin while inhibiting the expression of β-catenin, vimentin, twist, and snail (Figure 10L). In summary, the Erianin and 5-FU combination strategy enhanced the sensitivity of GC cells to 5-FU and prevented the oxidative stress in the liver caused by 5-FU. The combination also improved the inhibition of the EMT properties of tumors through the coordinated inhibition of the LKB1-SIK2/3-PARD3 axis.

## 3. Discussion

Gastric cancer is a highly malignant and aggressive type of digestive tract tumor that poses a significant threat to human health. In the search for effective anti-GC candidate compounds, traditional Chinese medicine (TCM) extracts present an area of immense potential. TCM is often perceived as a natural, low-toxic, and effective therapeutic approach. In this study, we demonstrated that Erianin, a bibenzyl compound extracted from dendrobium, could effectively inhibit the growth of GC cells both in vivo and in vitro and significantly inhibit the expression of LKB1-SIK2/3-PARD3. This may be one of the molecular regulatory basements of the EMT characteristics responsible for inhibiting GC cells. Furthermore, we also found that Erianin could be combined with 5-FU in vivo and in vitro to increase the sensitivity of GC cells to 5-FU.

EMT and metastatic spread of lesions are important steps in cancer progression. Characteristics of EMT include loss of polarity through downregulation of tight junctions and adherens junctions, as well as the remodeling of the extracellular matrix and cytoskeleton through upregulation of proteins such as fibronectin and vimentin [39]. PARD3 is a key regulator of epithelial cell polarity, and its dysregulation has been implicated in various malignancies. Studies have shown that PARD3 is highly expressed in B-cell malignancies, and its downregulation leads to increased cell apoptosis. In addition, the EMT regulatory factor snail is downregulated while the expression of E-cadherin is enhanced, highlighting the importance of PARD3 and snail-mediated EMT in metastatic tumors [40]. Similarly, in this study, we found that Erianin down-regulated the expression of PARD3 in GC cells while inducing apoptosis in GC cells, resulting in the upregulation of E-cadherin and the downregulation of β-catenin, vimentin, snail, and twist. As an essential component of the PAR complex that regulates the apical-basal polarity of epithelial cells, the role of PARD3 in EMT and tumor metastasis remains controversial, likely due to its context-dependent nature. Genome-wide screens have revealed that the gene encoding *PARD3* is missing in esophageal squamous cell carcinoma, lung cancer, and head and neck cancer cell lines [41,42]. In breast cancer, *PARD3* exists as a tumor-suppressor gene [43]. Interestingly, in ovarian cancer, a high expression of *PARD3* is associated with poor patient prognosis and may contribute to peritoneal metastasis [35]. In the context of GC, we found that PARD3 is highly expressed in the GC tissues of clinical patients. In the cell lines, we found that compared with gastric epithelial cells, PARD3 was highly expressed to varying degrees in four GC cell lines. Erianin down-regulated PARD3 while inducing GC cell apoptosis. To elucidate the regulatory mechanism of PARD3, we noticed that it encodes SIK, which was found to be involved in controlling the tight junctions of cells. Specifically, SIK1 can directly act on the polar protein PARD3 and induce its degradation [38]. However, the involvement of SIK in tumor metastasis remains controversial. It may act as a pro-metastasis factor or as a metastasis-inhibiting factor in the development of tumors. In orthotopic breast cancer models, reduced expression of the gene encoding *SIK1* is closely associated with the development of distant breast cancer metastasis [44]. On the contrary, *SIK1* is highly expressed in adrenocortical tumor cells of Y1 mice stimulated by adrenocorticotropic hormone [45]. In our study, we found that SIK2 and SIK3, but not SIK1, are responsible for the regulating PARD3 in GC cells. Both SIK2 and SIK3 were highly expressed in clinical patient tissues and GC cell lines, and the silencing of these kinases led to the inhibition of PARD3 expression. Previous studies have suggested that SIK2 is an important upstream regulator of carbohydrate response element-binding protein promoting the progression of liver cancer [46]. SIK3 expression is elevated in breast cancer cells and is associated with poor survival rates in breast cancer patients. Researchers used a combination of emodin and berberine to significantly inhibit SIK3 activity and reduce the proliferation of breast cancer cells [47]. These findings indicate that the inhibition of SIK2 and SIK3 may be an effective strategy for targeting the PARD3-mediated EMT and metastatic pathways in gastric cancer.

SIK is a serine/threonine kinase that belongs to the AMP-activated protein kinase (AMPK) family. One of the major upstream activators of AMPK is LKB1 [48,49]. SIK2 and SIK3 can be phosphorylated by LKB1 to play functions in cell metabolism, cell division, and cell metastasis [50]. A lack of LKB1 in cancer cells causes widespread metabolic changes, thereby promoting tumor initiation and progression. Therefore, LKB1 has long been considered a tumor-suppressor gene [51]. However, recent reports also suggest that LKB1 may have an unexpected role in promoting tumorigenesis. Studies have found that a high expression of cytoplasmic LKB1 is an independent marker of poor prognosis in breast cancer patients. The subcellular localization expression of LKB1 in breast cancer may serve as a prognostic biomarker [52]. In a paired study, it was reported that LKB1 expression is up-regulated in hepatocellular carcinoma (HCC) tissues relative to adjacent tissues and is positively correlated with the number of tumor lesions, tumor volume, and tumor grade. Down-regulation of LKB1 expression inhibits the proliferation of HCC cells. Therefore, researchers believe that LKB1 may be a proto-oncogene in HCC [53]. The researchers also discovered that ΔN-LKB1, a new isoform of LKB1, has intrinsic oncogenic properties. The silencing of ΔN-LKB1 reduces the survival rate of lung cancer cells NCI-H460 and inhibits their tumorigenicity when transplanted into nude mice [54]. In this study, we found that LKB1 is highly expressed in tumor tissues of clinical patients and is associated with poor prognosis of patients. Compared with GES-1, LKB1 expression in GC cell lines increased to varying degrees, and Erianin can inhibit the level of LKB1. This may be because Erianin has a better affinity with *LKB1* and inhibits its gene expression and protein translation.

In conclusion, this study reveal that Erianin may be a potential Dendrobium extract for the treatment of GC, which inhibits the growth and EMT of GC in vitro and in vivo through the LKB1-SIK2/3-PARD3 pathway. Furthermore, we found that this pathway is an important molecular basis for the combined inhibitory effect of Erianin and 5-FU on GC. Our results provide a pharmacological basis for treating GC by Erianin and a potential combined administration strategy for the treatment of GC. However, we still need more evidence to precisely determine the regulatory mechanism underlying Erianin’s effects in order to improve its transformation and application value.

## 4. Materials and Methods

### 4.1. Drugs and Reagents

Erianin was purchased from TopScience (T3864, Shanghai, China). Also, 5-fluorouracil (5-FU) was purchased from Sigma-Aldrich (F6627, Darmstadt, Hesse, Germany). Erianin (purity of 99.38%) and 5-FU (purity greater than 99%) were dissolved in dimethyl sulfoxide (DMSO) and stored at −20 °C. Before each drug treatment, the stock solutions were diluted with a culture medium to the desired concentration. In the corresponding experiments, a comparable amount of vehicle solvent (0.1% DMSO) was added to the control group. The following antibodies were obtained from various sources: β-actin (T0022), E-cadherin (AF0131), vimentin (AF7013), snail (AF6032), β-catenin (AF6266), LKB1 (AF6453), PARD3 (DF3368), and cleaved-caspase3 antibodies (AF7022) from Affinity Biosciences (Melbourne, Australia); Caspase3 (Cat. # 9662S), and PARP (Cat. # 9542S) antibodies from Cell Signaling Technology (Danvers, MA, USA); SIK2 (ab245211), and SIK3 (ab255701) antibodies from Abcam (Cambridge, UK); Twist (sc-6070) antibody from Santa Cruz Biotechnology (Dallas, CA, USA); And SIK1 (51045-1-AP) antibody from Proteintech (Chicago, IL, USA). Goat anti-mouse (ZB-2305) and goat anti-rabbit (ZB-2306) secondary antibodies, as well as the immunohistochemistry universal kit (mouse/rabbit polymer method detection system) (PV6000), were purchased from ZSGB-Bio (Beijing, China). Immunofluorescence secondary antibodies were obtained from Elabscience (Wuhan, China). All other reagents were purchased from standard commercial sources.

### 4.2. Clinical Patients

The fresh GC tumor samples used in this study were obtained from clinical patients. The study was approved by the Institutional Review Board of the First Affiliated Hospital of the University of Science and Technology of China (2023-Ky030), and informed consent was obtained from the patients. Patient data, including age, clinical diagnosis, clinicopathological characteristics, surgical records, and treatment methods, were collected for this study.

### 4.3. Cell Culture

The human GC cell lines SGC7901, BGC823, MGC803, and MKN45, as well as the human gastric mucosal epithelial cell line GES-1, were obtained from Shanghai Fuheng Biological Cell Bank (Shanghai, China). SGC7901 and MKN45 cells were cultured in Roswell Park Memorial Institute 1640 (RPMI 1640) medium supplemented with 10% (*v*/*v*) fetal bovine serum (FBS) (Gibco, Invitrogen, Carlsbad, CA, USA) and 1% penicillin-streptomycin (Biological Industries, Kibbutz Beit Haemek, Israel). BGC823, MGC803, and GES-1 cells were cultured in Dulbecco’s modified Eagle’s medium (DMEM) (Biological Industries, Israel) containing 10% FBS and 1% penicillin-streptomycin. All cell lines were stably cultured at 37 °C under standard environmental conditions containing 5% CO_2_.

### 4.4. Small Interfering RNA (siRNA) Transfection

RNA interference was performed in cells using specific siRNA. The siRNA oligonucleotide sequences are shown in Table 1. Cells were transfected with these siRNAs for 24 h using PepMute^TM^ siRNA Transfection Reagent (SignaGen, Frederick, MD, USA) [55]. The specific transfection procedures followed the instructions provided with the reagents. After 24 h, the cell lysates were collected and immunoblotting was performed with the corresponding antibodies to confirm the knockdown of the relevant target genes.

### 4.5. Molecular Docking

The 3D structure of Erianin and the protein structure of LKB1 (PDB ID: 2WTK) were downloaded from the PubChem database (https://pubchem.ncbi.nlm.nih.gov/, accessed on 9 January 2023) and the RCSB Protein Data Bank (PDB) database (https://www.rcsb.org/, accessed on 9 January 2023), respectively [56]. The online docking database CB-Dock2 (https://cadd.labshare.cn/cb-dock2/php/index.php, accessed on 9 January 2023) was used to perform molecular docking and obtain the best docking conformation and docking score. CB-Dock2 is a curvature-based cavity detection program and a molecular docking program of AutoDock Vina, which performs blind docking of protein ligands [57].

### 4.6. Gene Expression and Survival Curves

The *LKB1* gene was explored in the Cancer Genome Atlas (TCGA) dataset through the University of Alabama at Birmingham CANcer data analysis Portal (UALCAN) (https://ualcan.path.uab.edu/index.html, accessed on 12 October 2022) and investigation of genetic alterations associated with the cases of patients with gastric cancer [58]. The association of *LKB1*, *SIK2*, *SIK3*, and *PARD3* with the prognosis of gastric cancer patients was analyzed through the Kaplan–Meier Plotter database (https://kmplot.com/analysis/index.php?p=background, accessed on 12 October 2022) [59]. Patients were split by median and auto-selected the best cutoff.

### 4.7. Gene Correlation Analysis

GeneMANIA (https://genemania.org/, accessed on 15 November 2022) was used to gather a gene-gene interaction network of *LKB1* with *SIK1*, *SIK2*, *SIK3*, and *PARD3* to evaluate the association of these genes [60].

### 4.8. Cell Viability Assay

Cell viability was evaluated using Cell Counting Kit-8 (CCK8, Biosharp, Hefei, China) assay. Cells were seeded on 96-well plates at a density of 5 × 10^3^ cells/well. After the cells were subjected to the corresponding experimental treatment, 10 μL of CCK-8 reagent were added to each well. Following incubation for an appropriate duration at 37 °C, the absorbance at 450 nm was measured to quantify the reaction product.

### 4.9. Real-Time Quantitative PCR

Total RNA was extracted from tissue or cell samples using an RNA extraction kit (EZBioscience, Portland, OR, USA). The extracted RNA was reverse transcribed into cDNA using Color Reverse Transcription Kit (EZBioscience, Portland, OR, USA). SYBR Green qPCR Master Mix (EZBioscience, Portland, OR, USA) was used to perform qPCR reactions. The qPCR thermocycling program consisted of: hot-start enzyme-activation step at 95 °C for 5 min, followed by 40 cycles of denaturation at 95 °C for 10 s and combined annealing and extension at 60 °C for 30 s. The relative mRNA expression levels of target genes were normalized using *β-actin* as an internal control. The primer sequences used in the qPCR analysis were referenced from previous studies [61,62,63,64,65] and are provided in Table 2.

### 4.10. Western Blot

The treated cells or fresh tissues were collected on ice and lysed thoroughly in RIPA lysis buffer (Beyotime, Shanghai, China) supplemented with a protease and phosphatase inhibitor cocktail (Beyotime, Shanghai, China). After centrifugation at 4 °C, the supernatant was collected and denatured by adding loading buffer (Beyotime, Shanghai, China). The samples were then separated by SDS-PAGE and transferred onto a PVDF membrane (Merck Millipore, MA, USA). The membrane was blocked with 5% skim milk for 1 h and incubated with the corresponding primary antibody overnight at 4 °C. Subsequently, the membrane was incubated with the appropriate secondary antibodies for 1 h at room temperature. The protein bands were visualized using a chemiluminescence detection system (Biosharp, Hefei, China). Quantitative analysis of protein bands was completed using ImageJ software (version 1.48).

### 4.11. Hoechst 33342 Staining

GC cells were seeded into a six-well plate containing glass slides at a density of 5 × 10^5^ cells/well. The following day, the cells were treated with 0, 50, 100, 200, and 400 nM of Erianin and cultured for 24 h. Hoechst 33342 staining solution (Beyotime, Shanghai, China) was then added to cover the cells, and the cells were stained for 3–5 min in the dark [66]. Subsequently, the cells were washed two-to-three times with PBS for 3–5 min each time. Finally, an appropriate amount of anti-fluorescence quenching mounting medium was added to the cells, and the samples were observed under a fluorescence microscope (Olympus, Hachioji City, Tokyo, Japan).

### 4.12. Wound-Healing Assay

GC cells were seeded in six-well plates with a density of 5 × 10^5^ cells/well. When the cells reached near-confluency, a sterile 200 µL pipette tip was used to create a scratch in the cell monolayer, simulating a wound. The detached cells were then removed by washing with PBS, and fresh culture medium was added to continue the incubation. The GC cells were treated with different concentrations of Erianin. The scratch wounds were observed, and images were captured under an inverted phase contrast microscope at 0 and 24 h. The wound surface area was calculated using ImageJ software [67].

### 4.13. Migration and Invasion Assays

GC cells were seeded in a six-well plate at a density of 5 × 10^5^ cells/well. The following day, the cells were treated with corresponding concentrations of Erianin for 24 h. Cells from the respective treatment groups were then harvested by digestion and a total of 5 × 10^4^ cells in 200 µL FBS-free medium were seeded into the upper chambers of transwell inserts (Corning, Corning, NY, USA) without or with pre-coated matrigel. The lower chambers were filled with 500 µL of culture medium containing 10% (*v*/*v*) FBS. Then, cells were placed in a 37 °C culture phase containing 5% CO_2_ and continued to be cultured for 24 h. Afterward, cells were fixed with 4% paraformaldehyde (Biosharp, Hefei, China) for 15 min and stained with 0.1% crystal violet staining solution (Biosharp, Hefei, China) for an appropriate time. The stained cells were then imaged under a microscope [67].

### 4.14. Immunofluorescence

GC cells were seeded in six-well plates containing glass slides. After the cells were treated accordingly, the culture medium was discarded and the cells were gently washed twice with PBS. The slides were then fixed with 4% paraformaldehyde for 15 min. Following fixation, the slides were washed twice with PBS, and the cells were permeabilized with 0.5% Triton X-100 for 15 min. Next, the cells were blocked with 5% bovine serum albumin (BSA) for 1 h. The cells were then incubated overnight at 4 °C with LKB1 antibody (1:100). Next daylight period, the cells were incubated with green fluorescent secondary antibody (Elabscience, Houston, TX, USA) for 1 h at room temperature in the dark. Afterward, the cells were incubated with DAPI (Biosharp, Hefei, China). Finally, an appropriate amount of anti-fluorescence quenching mounting medium (Beyotime, Shanghai, China) was added dropwise, and the slides were observed under a fluorescence microscope [67].

### 4.15. Animal Experiment

The animal study was approved by the Animal Ethics Committee of the First Affiliated Hospital of the University of Science and Technology of China (2023-N(A)-24). Thirty male BALB/c nude mice with weight 20 ± 2 g were obtained from Hangzhou Ziyuan Experimental Animal Technology Co., Ltd. (Hangzhou, China) and were adaptively raised in an SPF environment for 3 days. MKN45 cells suspended in PBS were subcutaneously injected into the right armpit of nude mice (3 × 10^5^ cells/mouse). When the average tumor volume in the mice reached 100 mm^3^, the mice were randomly divided into the following groups, with six mice in each group: vehicle control group, 5-FU group (50 mg/kg/3d) [68], low dose of Erianin group (10 mg/kg/d), medium dose of Erianin group (20 mg/kg/d), and high dose of Erianin group (50 mg/kg/d) [69]. In the animal experiment involving the combination of Erianin and 5-FU, the following groups were included, with six mice in each group: vehicle control group, Erianin group (20 mg/kg/d), 5-FU group (25 mg/kg/3d), and Erianin (20 mg/kg/d) + 5-FU (25 mg/kg/3d) group. The dissolution procedure for Erianin is 10% (*v*/*v*) DMSO + 40% (*v*/*v*) PEG300 + 5% (*v*/*v*) Tween-80 + 45% (*v*/*v*) saline. The dissolution procedure for 5-FU is 5% (*v*/*v*) DMSO + 30% (*v*/*v*) PEG300 + 5% (*v*/*v*) Tween-80 + 60% (*v*/*v*) ddH_2_O. All drug treatments were administered intraperitoneally and lasted for 12 days. During the experimental period, the mice’s body weight and tumor volume were measured and recorded daily. Tumor volume was calculated according to the formula V = 1/2 (width^2^ × length). At the end of the experimental, the mice were sacrificed, and the xenograft tumors, spleens, livers, and kidneys were harvested for further analysis. The liver, spleen, and kidney indexes were calculated as based on the Organ index = Organ weight (g)/body weight (g).

### 4.16. Immunohistochemistry (IHC)

Human GC tissue, normal tissue, or mouse tumor tissue slides were prepared for immunohistochemical analysis. The slides were baked at 65 °C for 30 min, then dewaxed in xylene twice, with each step lasting 10 min. Next, the slides were soaked in absolute ethanol twice, again with each step lasting 10 min. The sections were then hydrated through a graded ethanol series (95%, 85%, and 75%) for 5 min each. The sections were washed twice in distilled water for 5 min each, then permeabilized with 0.5% Triton X-100 for 30 min. After that, the sections were treated with an endogenous peroxidase blocker for 10 min. Antigen retrieval was performed using a heated sodium citrate antigen retrieval solution (1×) for 3 min. The sections were then blocked with 5% BSA for 1 h. The sections were incubated with primary antibodies against LKB1, SIK2, SIK3, and PARD3 overnight at 4 °C. Subsequently, the sections were incubated with enzyme-labeled goat anti-mouse/rabbit IgG polymer for 1 h at 37 °C. The color of sections was developed using DAB substrate kit and then counterstained with hematoxylin. Finally, the slides were dehydrated through a graded alcohol series and covered with coverslips. The staining intensity of LKB1, SIK2, SIK3, and PARD3 in the sections of each group was observed under a microscope.

### 4.17. Hematoxylin and Eosin (H&E) Staining

The tissue sections were first dewaxed and hydrated. They were then immersed in 85% and 95% ethanol solutions for 5 min each. Next, the sections were stained with eosin stain (alcohol-soluble) for 5 min. The stained sections were then dehydrated by immersing them in absolute ethanol three times, with each step lasting 5 min. Using a pipette, 100 μL of a pre-prepared hematoxylin staining solution was added dropwise to each tissue section, and the sections were stained for 10 min. After the hematoxylin staining, the excess staining solution was washed away using distilled water. The sections were then cleared in xylene for 5 min, repeating this step once more. Finally, the slides were sealed with neutral gum, and images of the stained sections were captured under a microscope.

### 4.18. Superoxide Dismutase (SOD) and Malondialdehyde (MDA) Assay

Liver tissue samples were collected from mice in each experimental group. An appropriate amount of liver tissue was added to RIPA lysis buffer at a ratio of 100 mg tissue per 1 mL of lysis solution. The tissue samples were then thoroughly lysed using a tissue homogenizer. The protein content of the tissue lysates was measured using a BCA protein concentration assay. The relative levels of SOD and MDA in the mouse liver tissue were determined using commercial assay kits. Specifically, the SOD activity was measured by SOD assay kit (Beyotime, Shanghai, China) and lipid peroxidation level, as indicated by MDA, was assessed using a lipid peroxidation MDA assay kit (Beyotime, Shanghai, China) [70,71]. All experiments were conducted strictly according to the manufacturer’s recommended protocols.

### 4.19. Statistical Analysis

All data were collected in triplicate and expressed as mean ± SD. The data analysis was performed using GraphPad Prism 6 software (La Jolla, CA, USA). T-test performed statistical comparisons between the two groups. Multiple comparisons were performed using nonparametric one-way ANOVA or two-way ANOVA. The sample data obtained from the TCGA database were analyzed using R language. For the Kaplan–Meier survival analysis, the log-rank test (Mantel–Cox) was used to assess statistical significance. The Benjamini–Hochberg method was employed to correct for multiple hypothesis testing and calculate the adjusted *p* value. *p* < 0.05 was considered statistically significant.

## 5. Conclusions

In summary, Erianin has a pro-apoptotic effect on GC cells. By down-regulating LKB1, Erianin reduces the expression of its downstream SIK2 and SIK3, thereby reducing the level of PARD3 and inhibiting the migration and invasion abilities of GC cells and its tumorigenesis in vivo. 5-FU has a certain inhibitory effect on LKB1, which may be the basis of its combined effect with Erianin. The combination of Erianin and 5-FU increases the sensitivity of GC cells to 5-FU and enhances its anti-tumor effect in vivo. Moreover, Erianin has good biological safety and may serve as a promising LKB1 inhibitor and candidate drug for treating gastric cancer.

## Figures and Tables

**Table 1:** siRNA sequence.

siRNA Primer	Sequence (5′-3′)
*siLKB1*-1-F	GGGCCAAGCUCAUCGGCAATT
*siLKB1*-1-R	UUGCCGAUGAGCUUGGCCCTT
*siLKB1*-2-F	UGUAUAUGGUGAUGGAGUATT
*siLKB1*-2-R	UACUCCAUCACCAUAUACATT
*siLKB1*-3-F	GGGACAACAUCUACAAGUUTT
*siLKB1*-3-R	AACUUGUAGAUGUUGUCCCTT
*siSIK2*-1-F	GGUAUGUCCCGGUGAAUUATT
*siSIK2*-1-R	UAAUUCACCAGGACAUACCTT
*siSIK2*-2-F	UUGCAGAACAAGAGCUAUATT
*siSIK2*-2-R	UAUAGCUCUUGUUCUGCAATT
*siSIK2*-3-F	GGCUAGAACCAAAGGAAUUTT
*siSIK2*-3-R	AAUUCCUUUGGUUCUAGCCTT
*siSIK3*-F	GUAGAAUGGCAGAAAAGGATT
*siSIK3*-R	UCCUUUCUGCCAUUCUACTT
*siPARD3*-1-F	GAGAUAAGGAGAAGGAUAATT
*siPARD3*-1-R	UUAUCCUUCUCCUUAUCUCTT
*siPARD3*-2-F	GGGCAAAUCCCAAGAGGAATT
*siPARD3*-2-R	UUCCUCUUGGGAUUUGCCCTT
*siPARD3*-3-F	ACAUGGAGAUGGAGGAAUATT
*siPARD3*-3-R	UAUUCCUCCAUCUCCAUGUTT
NC-F	UUCUCCGAACGUGUCACGUTT
NC-R	ACGUGACACGUUCGGAGAATT

**Table 2:** Gene sequences (human).

Gene Primer	Sequence (5′-3′)
*LKB1*-F	TGTCGGTGGGTATGGACAC
*LKB1*-R	CCTTGCCGTAAGAGCCTTCC
*SIK1*-F	GAGTCACCAAAACGCAGGTTG
*SIK1*-R	AGTGACGATGTAAAGCATGTCC
*SIK2*-F	TGAGCAGGTTCTTCGACTGAsT
*SIK2*-R	AGATCGCATCAGTCTCACGTT
*SIK3*-F	TCAGCAGCAACCTGAGAACT
*SIK3*-R	ACAAGGGACGGTGCCCATAG
*PARD3*-F	CAGACAGAACTACTAACTTCGCC
*PARD3*-R	ATGC CTCGGATGAAGAGTCCT
*β-actin*-F	CTCACCATGGATGATGATATCGC
*β-actin*-R	AGGAATCCTTCTGACCCATGC

## Data Availability

The data supporting the results of this study are available from the corresponding author upon request.

## Abbreviations

AMPK: AMP-activated protein kinase; BSA: bovine serum albumin; CDX: cell line-derived xenograft; DMEM: Dulbecco’s modified Eagle’s medium; DMSO: dimethyl sulfoxide; EMT: epithelial-mesenchymal transition; FBS: fetal bovine serum; GC: gastric cancer; HCC: hepatocellular carcinoma; H. pylori: helicobacter pylori; LKB1: Liver kinase B1; MDA: malondialdehyde; MDR1: multidrug resistance-related protein 1; NSCLC: non-small cell lung cancer; PARD3: Partition defective 3; PDB: Protein Data Bank; RPMI 1640: Roswell Park Memorial Institute 1640; SIKs: Salt-inducible kinases; siRNA: Small interfering RNA; SOD: Superoxide dismutase; STK11: serine/threonine (Ser/Thr) kinase 11; TCGA: the Cancer Genome Atlas; UALCAN: University of Alabama at Birmingham CANcer; 5-FU: 5-fluorouracil.
